# Analysis of Selected Properties of Welded Joints of the HSLA Steels

**DOI:** 10.3390/ma13061301

**Published:** 2020-03-13

**Authors:** Ivan Miletić, Andreja Ilić, Ružica R. Nikolić, Robert Ulewicz, Lozica Ivanović, Norbert Sczygiol

**Affiliations:** 1 Faculty of Engineering, University of Kragujevac, 34000 Kragujevac, Serbia; gilic9@sbb.rs (A.I.); lozica@kg.ac.rs (L.I.); 2 Research Center, University of Žilina, 01026 Žilina, Slovakia; ruzica.nikolic@rc.uniza.sk; 3 Department of Production Engineering and Safety, University of Technology, 42201 Częstochowa, Poland; robert.ulewicz@pcz.pl (R.U.); norbert.sczygiol@pcz.pl (N.S.)

**Keywords:** high-strength low-alloyed steels, impact toughness, micro hardness, V-butt multiple-pass weld, MMA/MAG welding procedure(s)

## Abstract

This paper presents research of the impact toughness and hardness distribution in specific zones of a ‘single V’butt multiple-pass welded joints of the high-strength low-alloyed steels. Obtained values of the impact toughness are analyzed in correlation with a microstructure in specific zones of the welded joint, together with the micro hardness distribution found in the related zones. Based on the carried out analysis and results obtained in experiments, the applied technology of welding was evaluated. The original conclusions on influence of the selected welding procedure manual metal arc (MMA) for the root passes and metal active gas (MAG) for the filling and covering passes) on impact toughness of the high-strength low-alloyed steels are drawn. The paper also presents discussion on the valid standards and recommendations related to welding of those steels, from the aspect of applications in design of steel welded constructions.

## 1. Introduction

Joints formedby welding, especially in the case if parts made of the high-strength low-alloyed steels (HSLA) being joined, stands for a complex process with a large number of influential factors and with their complex mutual interactions that additionally complicate the process itself. Due to the significance and specificity of welding, high-strength low-alloyed steel producers prescribe the relevant welding technology steels they provide. The HSLA steels can be considered as conditionally weldable, whereby this conditionality refers to application of measures that provide for the successful joining by welding. In the process of welding, these steels suffer degradation of mechanical properties due to changes of their microstructural state. Those changes are especially significant in the heat-affected zone (HAZ) of the welded joints, such as increase of stiffness, decrease of toughness, increase of brittleness due to ductile transition temperature, as well as appearance of different kinds of material discontinuities. Appearance of cracks and the brittle phases is related to the fast cooling of the butt weld and its surrounding, within the temperature limits characterized by the largest instability of austenite. Due to the presented specifics of this steel grade, it is not sufficient only to take care of technological welding parameters that provide for the proper weld formation, but it is necessary to consider all the aspects of applied technology, as well as its negative effects altogether with sensitivity to those effects.

Considerations of the welded joints of the HSLA steels require pointing to the following: limited plasticity reserve, high tensile strength, possibility of local zones of the lower plasticity as compared to the rest of the construction, as well as the possibility of appearance of cold cracks in the weld metal and the HAZ, which can be potential initiation spots for brittle fracture.

Luo et al. [1] analyzed the influence of morphology of martensite-austenite constituents on impact toughness of the HSLA steels in the inter-critically reheated coarse-grained heat-affected zones. The three different two-pass weld thermal cycles were applied by using the thermal simulator base metal of a HSLA steel, for the purpose of obtaining the martensite-austenite constituents of different morphologies. The authors concluded that slender constituents are more harmful to toughness than the massive ones. Taban, Kaluc, and Kocaeli [2] have considered application of the super-martensitic stainless steels (SMSS) as efficiently and effectively selected materials for fabrication of onshore and offshore pipelines. They also emphasized that fatigue loading behavior of welded structures, made of the super-martensitic stainless steels, has not been thoroughly investigated.

Chen et al. [3] presented an experimental studyof welding effects on tensile characteristics of the T-welds of the high-strength low-alloyed steel. Values of mechanical characteristics in the elastic zone, obtained experimentally, are higher than the related values calculated according to Eurocode 3 standard: steel constructions design [4], implying that the standard prescribes determination of the discussed weld characteristics of the HSLA steel in a conservative way.Zhang et al. [5] presented experimental investigation of the welding influence on strength of the high performance steels and concluded that welding significantly softened the heat affected zone in the HSLA steel with insignificant effect on the normal strength.

Durmusogly, Turker, and Tosun [6] were investigating gas metal welding (GMA) of the HY-80 steels with application of different welding parameters. They subjected different samples, obtained from the welded joints, to tensile, hardness, and impact toughness tests. The measured values of hardness in the weld metal were almost similar to the base metal values, while the hardness values in the HAZ were higher. Saedi, Hajjari, and Sadrossadat [7] presented an analysis of microstructural characteristics and mechanical properties of the dissimilar joining weldments of the API 5L X60 ferritic HSLA steel and the AISI 310S austenitic stainless steel, by using theoptical and scanning electron microscopy, ferritometry, micro hardness, tensile, and impact tests. Verma and Taiwadem [8] showed that joining of duplex alloys is challenging due to number of embrittling precipitates and metallurgical changes, while inappropriate welding conditions and unbalanced phase ratio of austenite/ferrite, lead to solidification cracking, corrosion susceptibility, and lower ductility. Authors also presented a systematic review and highlighted the effects of welding on microstructure, mechanical properties and corrosion resistance of duplex stainless steels.

Hosseinioun et al. [9] were performing the multi-run welding of the HSLA steel plates executed by the MMA procedure. They emphasized that it is essential to quantify the weld microstructure after each inter-run, as well as its constitution, since that is what influences the final mechanical properties of the welded structures. Their analysis of the weld metal microstructures revealed the following: “(1) in the last run, there were intergranular acicular ferrite and pro-eutectoid ferrite, (2) in previous weld runs, there were refined grains with precipitated particles and (3) in the interlayer region of previous runs, there were some heterogeneous ferrite grains, grain boundary discontinuities and precipitated micro-alloying elements”. Dundjer et al. [10] presented research of influence of the welding parameters on the HSLA steel and on hardness and impact energy of samples welded at related different welding parameters. Saxena et al. [11] presented a comparative study of the tensile and impact properties of the multi-pass SMAW Armox 500T steel joints, executed with the two different consumables: austenitic stainless steel and low hydrogen ferritic steel. The research presented in that paper pointed out that the joint efficiency of weldment with the low hydrogen ferritic steel consumable was 41.7%, while for weldment with austenitic stainless steel consumable it was 30.6% of its base metal. Those facts indicated influence of the electrode-consumable on tensile properties of the welded joints.

Haslberger et al. [12] have presented a research of the multi-pass welded joints of the high-strength low-alloyed steel, done by the underwater local cavity welding and underwater dry welding techniques. The micro-hardness of the HAZ and fusion zone in the underwater local cavity welding specimen was higher than in the related zones in the underwater dry welding joint. The yield strength and ultimate tensile strength values of the underwater local cavity welding specimens were lower than values of those properties of the underwater dry welding joint. However, the impact toughness of the underwater dry welding specimens was much higher than that of the underwater local cavity welding specimens. Alipooramirabad et al. [13] implied that one of the important steps in design and fabrication of welded structures is selection of the welding process and the filler consumables. Furthermore, it is concluded that this is because those two factors are controlling the mechanics of thermal distribution and the chemistry of the weld. Experimental results showed that the higher level of residual stresses was registered at the modified short arc welding and the flux cored arc welding in accordance with the microstructural and mechanical properties. The higher levels of residual stresses may be related to formation of bainite and Widmanstätten ferrite in the weld metal and HAZ.

Mechanical characteristics of the HAZ of the HSLA steel weld are dominant to both resulting mechanical characteristics and mechanical behavior of the welds on tests during exploitation according to Xue [14], Ilić [15], Ilić et al. [16], and Ivanović et al. [17]. Mechanical characteristics, in correlation with evaluation of microstructure in specific zones, as well as analysis of the micro hardness distribution are the basis for design of welded constructions made of the HSLA steel. Full advantages provided by the related material usage can be achieved only by this design perspective, Ilić [15].

Zhanget al. [18] presented an analysis of the microstructure effect on mechanical properties of the high-strength low-alloyed steel during the welding with/without additional layers (buffer layers). Influence of the HAZ characteristics on overall characteristics of the welds for the low-carbon steels is analyzed by Gharibshahiyan et al. [19], due to the fact that this zone is characterized by altered microstructure and the high level of residual stresses. This work analyzes effect of the welding parameters, i.e., the heat input, on the growth rate of the metal grains and their ratios. Szataniak, Novy and Ulewicz [20] were considering use of different cutting techniques for HSLA steels. They realized that mechanical cutting works for thin sheets made of structural steel. For thicker sheets, different technologies of oxygen cutting, plasma, laser, and water jet cutting must be applied. Bokůvka et al. [21] were studying influence of the welded joints’ quality on safety and reliabilityin operation. Despite the usual assumption that the welded joints of steels possess long-term reliability, they can be causes of fracture and even crashes of plants due to defective work of bad quality of joints’ execution. Nový et al. [22] presented selected experimental results of investigation of the structural steels fatigue resistance. They performed experiments on nine structural steels including high strength steels, DOMEX 700MC, HARDOX 400, HARDOX 450, 100Cr6 (Ultimate Tensile Strength (UTS) from 446 to 2462 MPa) at high-frequency cyclic loading (f = 20 kHz, T = 20 ± 5 °C, R = −1) in the range of the loading cycles numbering from 2×10^6^ to 2×10^9^. They observed the continuous decrease of fatigue strength in dependence on the number of loading cycles, with the average value of ratio (σ_a_ 2×10^9^)/(σ_a_ 2×10^6^) = 0.69.

Since the cooling rate is variable and decreases with the temperature decrease, the cooling time from 800 to 500 °C (t_8/5_) is usually taken as the parameter that the best characterizes the cooling conditions of the HAZ, with respect to the lowest stability of austenite of the majority of steels. The cooling time in that temperature range can be calculated according to different expressions, as well as determined experimentally. Knowing that cooling time and relevant material properties, enables prediction of characteristics of the welded joint and the heat affected zone [23,24,25].

Compared to conclusions of the presented references, this paper presents an analysis of the welding effects on impact toughness of the high-strength low-alloyed steels of one of the most commonly used welding procedures in present industry. The research further establishes the basic qualitative relations between the pre-existing microstructure, relevant micro hardness values, and measured values of the impact toughness. Derived conclusions can be used for both the relevant technology and welding parameters identification of this steel grade, as well as a basis for the future detailed research related to quantifying of certain effects established qualitatively in this research.

## 2. Experimental Testing

**Figure 1:**
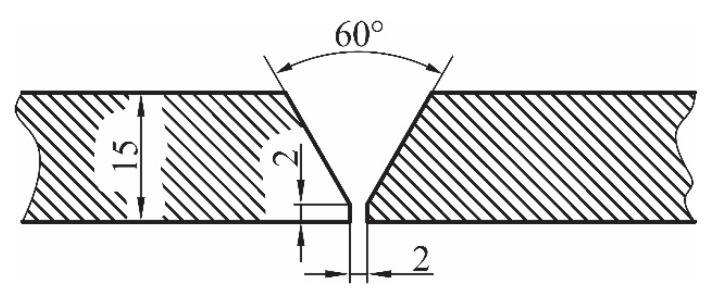
Shape and dimensions of the single V groove.

Experimental testing is done on the models made of the high-strength low-alloyed steel that is classified according to standard EN 10025–6:2004 [26] as class S690 and meets defined properties and conditions. The chemical composition of the considered steel is given in Table 1. Experimental testing samples are made by the multiple-pass V-groove butt weld joint at the central zone. The root passes of considered welds are executed by the manual metal arc (MMA) procedure. The filling and covering passes are made by the metal active gas (MAG) welding procedure in the gas compound (82% Ar + 18% CO_2_). Before the welding, it was necessary to prepare the groove. Shape and dimensions of the welding groove is presented in Figure 1, while parameters and related consumables of the welding procedures are presented in Table 2.

**Figure 2:**
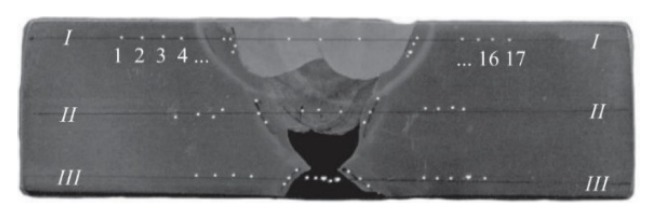
Cross-sections of a metallographic sample with directions and micro hardness measuring spots.

Metallographic analysis of the welds with measuring of micro hardness is done in three parallel directions in the cross-sections of prepared metallographic samples. The cross-section of the metallographic samples with directions and micro hardness measuring spots is presented in Figure 2.

Directions of the analysis and micro hardness measuring spots are set in characteristic zones in metallographic samples cross-sections, so that they run through the parent metal, heat-affected zone and the weld metal: I-I is on the face of the weld; II-II is in the central zone with interfered impacts of the root and other passes and III-III is on the side of the root pass. Further assessment of the pre-existing microstructure state is done by the reflex optic micrograph method at enlargements of 200× and 500×. Basic chemical and mechanical characteristics of used consumables are shown in Table 3.

The used filler metals marks by EN codes and usual applications are as follows:

(1) INOX B 18/8/6, mark EN 18 8 Mn B 22 according to EN 1600–Applied as interlayers for application of the root welds aimed to decrease the residual stresses and increase the weld plasticity and toughness.
(2) MIG 75, mark Mn3Ni1CrMo according to EN 12534–For fine grains HSLA steel welding with yield stress up to 690 MPa.

**Figure 3:**
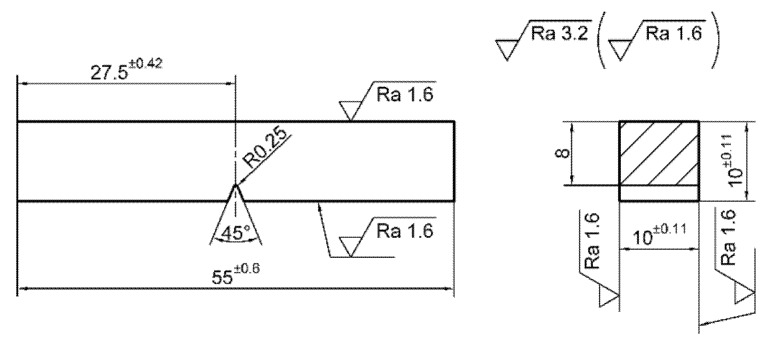
Impact toughness test samples, shape and dimensions.

During the following stage, experimental testing of impact toughness and analysis of mechanical behavior during the fracture were done on the series of three test samples. Shapes and dimensions of the test samples for the impact toughness testing of type ISO*-V* (55 × 10 × 10) are shown in Figure 3.

The testing procedure, shape and dimensions of the test samples were in compliance with adequate standard [27]. The pendulum speed for the impact toughness test was 5 to 5.5 m/s, while the energy loss was less than 1%. Experimental testing was carried out at room temperature.

The time between the interpasses is measured until the temperature of the deposited layer does not drop below 225 °C and it amounts to between 3 and 5 min.

## 3. Results and Discussion

**Figure 4:**
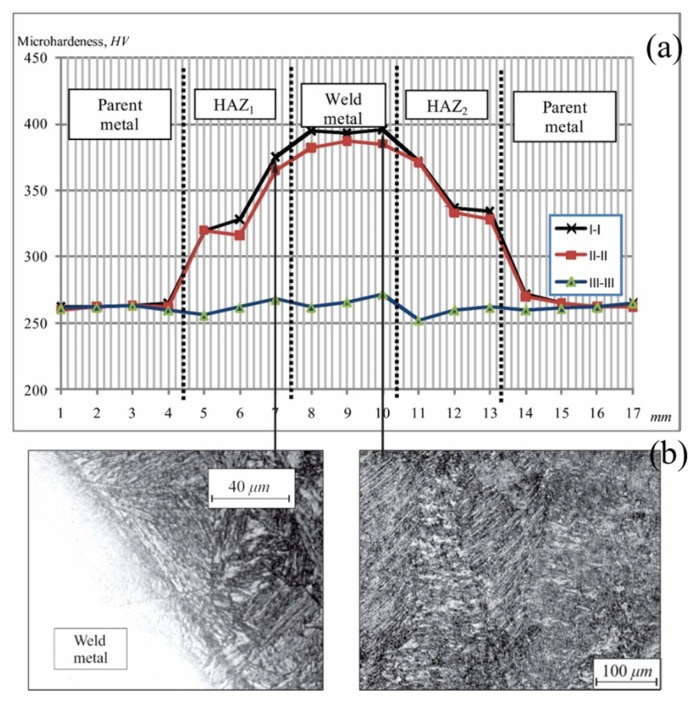
Micro hardness distribution and micro-structure of samples: (**a**) zone of transition between the weld metal and HAZ; (**b**) weld metal.

The micro hardness distribution along all the three considered directions, as well as micro-photography of the pre-existing microstructure states for the MMA/MAG welds are presented in Figure 4.

According to diagram of the micro hardness distribution in all directions, it is evident that the micro hardness measured values do not exceed the critical values of the brittle structures formation what is in accordance with the fact that no brittle structures of microstructural states are recorded in the micrographic analysis. The critical values, prescribed by standard are from 380 to 390 HV. The fine-grain and coarse grain zones are present in the HAZ along all the directions and are common for a large number of welding procedures by melting. In direction I-I the coarse grain bainite microstructure with quenched martensite is registered at the spots close to the fusion zone. In the HAZ, close to the parent metal, a favorable fine-grain ferrite-pearlite structure with bainite is registered. The slightly higher micro hardness is registered in the weld metal as compared to the micro hardness measured in the HAZ, while the microstructure is assessed as the coarse grain bainite with a higher share of martensite, which is in correlation with measured micro hardness values, Figure 4b. The measured micro hardness values in direction II-II are lower than those in specific zones in direction I-I and do not exceed the critical values relevant for the brittle microstructural states. The lower micro hardness values result from the microstructure relief due to the heat input from the filling passes of the multiple-pass welding. The coarse grain microstructure of the HAZ in direction III-III of metallographic samples, obtained by the MMA/MAG welding procedure, is bainite without bold acicular structures. The root pass metal weld remained non-erosive during the preparation of metallographic samples due to the high-alloyed filler material used for the root pass, as presented in Figure 4a. The transient zone between the weld metal and the HAZ is characterized as bainite microstructure without acicular tendency in compliance with the lowest micro hardness measured in this zone, as compared to the related values in direction II-II.

**Figure 5:**
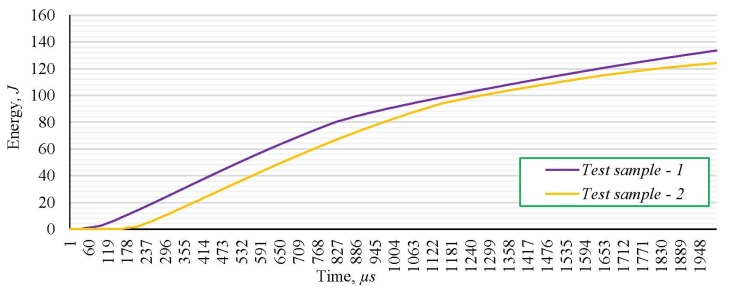
Flow of energy during the impact toughness test of the S690 samples.

**Figure 6:**
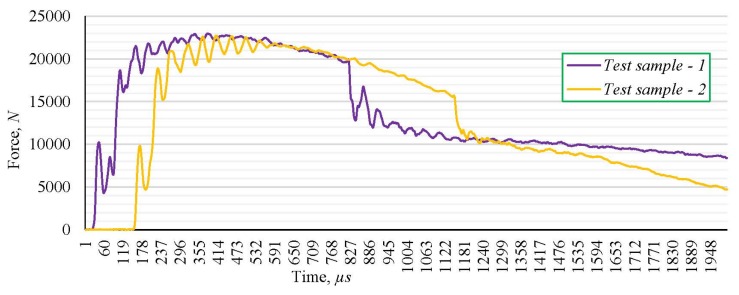
Force-time dependence during the impact toughness testing of the parent metal.

The energy flow during the fracture of the two test samples, marked as 1 and 2, without the weld joint, at impact toughness testing, are presented in Figure 5, while the force variation during the testing of samples, made of the parent metal without a welded joint, is presented in Figure 6.

Experimentally obtained values of the energy, as well as force–time dependence during the fracture of the tested samples, demonstrate insignificant variations for two different samples and the character complying with the standard; accordingly, obtained results can be used as relevant for further analysis. Based on the experimentally obtained values of the fracture energy and impact toughness, it can be concluded that the considered steel completely meets prescribed standard values at room temperature. The average percentage of experimentally obtained ductile fractures is 57.83%, whichimplies the tough mechanical behavior of samples made of steel S690QL during the testing at room temperature (Figure 5 and Figure 6). Appearance of the fracture surface implies the mixed fracture type with the large percentage of ductile fracture, whichcorrespondswithexperimentally obtained results.

**Figure 7:**
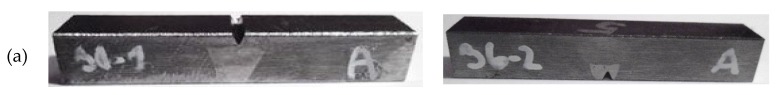

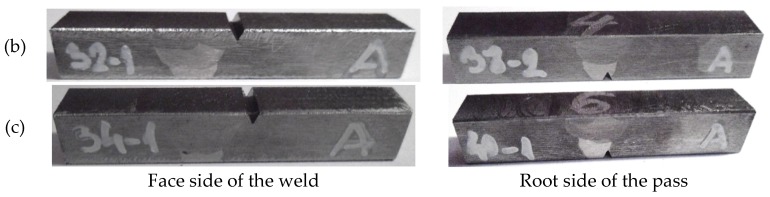
Positions of the notch on samples for the impact toughness testing: (**a**) Along the weld metal axis; (**b**) in the fusion zone; (**c**) in the HAZ.

Experimental testing was then done on the prepared test samples that contain considered welded joints with a notch positioned along the weld axis, then in the zone of fusion and at the end in the HAZ on the face and on the root pass side. Characteristic zones of the weld became visible by chemical etching with a 4% nital solution, which enabled precise positioning of the notch. The notch positions on test samples with the welded joint are presented in Figure 7. Testing procedure, shape and dimension and characteristics of the used testing pendulum are the same as for testing of samples without the welded joint that is previously described in details.

**Figure 8:**
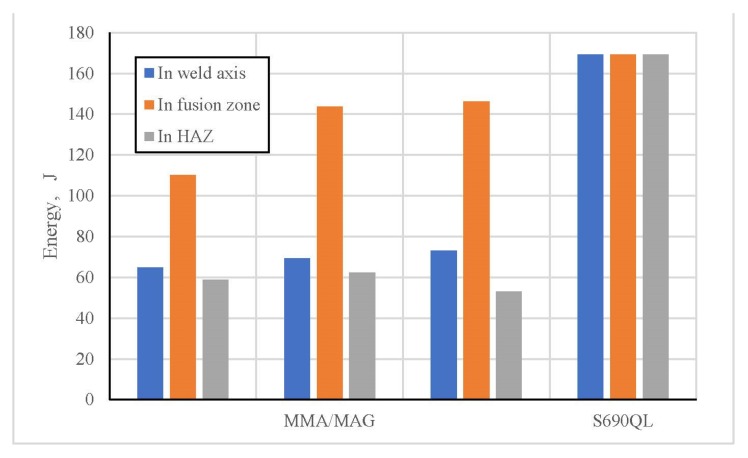
Fracture energy histogram of the test samples with a notch on the face side of the welds.

**Figure 9:**
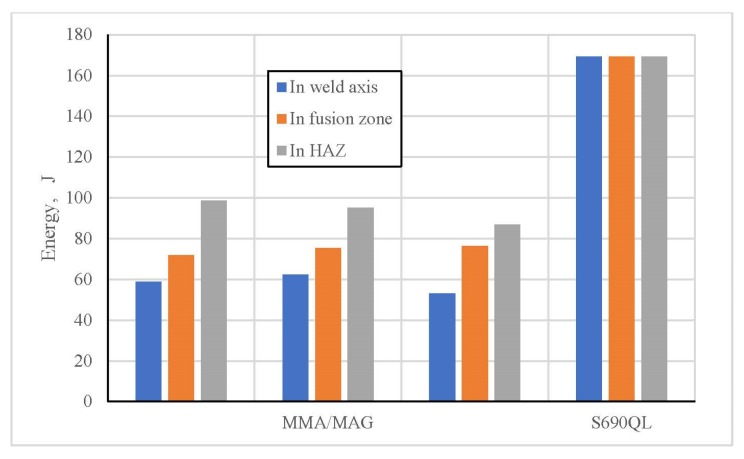
Fracture energy histogram of the test samples with a notch on the root side of the pass.

Experimentally obtained values of the fracture energy of the test samples with a notch on the face side of considered welds are presented in Figure 8, while for the test samples with a notch on the root side they are shown in Figure 9.

**Figure 10:**
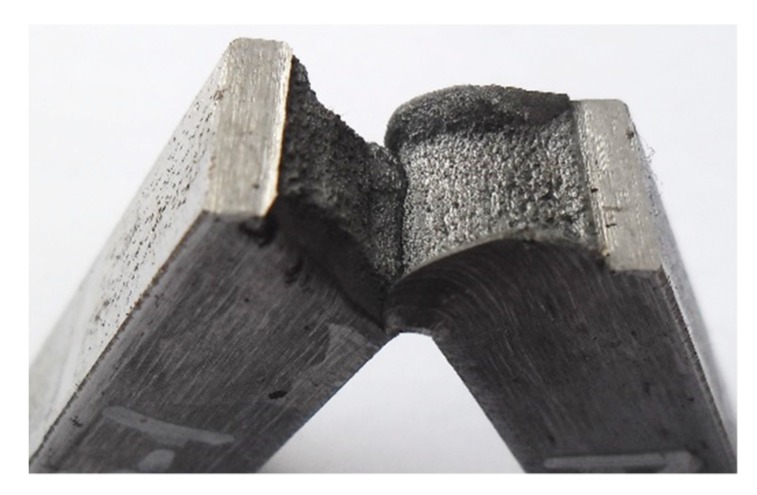
Fracture surface of the test sample with a notch in the HAZ on the face side of the weld.

Presented results of the fracture energy are completely in correlation with prior existing microstructure state, as well as with the corresponding micro hardness values. Along with presented experimental procedures, the visual assessment of the fracture surface characteristics was done. This implied the mixed character of fracture and that is in accordance with the above mentioned facts. The fracture surface of the test samples with a notch on the face side in the HAZ, done by the MMA/MAG welding, is presented in Figure 10.

Values of the cooling time t_8/5_ were obtained within range 28.5 to 32 s. Different theoretical formulas predict that values could range from 24 s (Ito-Bessyo formula) to 65 s (formula of limited thickness) [25], so the experimentally obtained values are in compliance with theoretically predicted ones.

## 4. Conclusions

The high-strength low-alloyed steels present a significant steel grade of a general purpose with specific properties, under conditions when the welding procedures stand for a method of joint execution. Application of this steel grade in the welded constructions can be seen from the two aspects: mechanical reaction in the exploitation conditions and technological aspect of welding application for the joints making. The welding plan combined the two different procedures of obtaining the welded construction. Selected procedures represent welding procedures ordinarily used in industrial practice. Mechanical characteristics and response to load during the testing of the impact toughness point to a significant deterioration of mechanical properties of observed steel S690QL, due to applied welding procedures. Microstructures of specific zones of welded joints, assessed by metallographic methods, were expected and are in agreement with theoretical observations.

The samples with the welded joints did express the termination zones of the fast crack growth during the testing.

Experimentally obtained values of the fracture energy of those samples was smaller than values obtained for samples without the welded joints, made of the parent metal. This confirms the fact that welding causes a decrease of the fracture energy of the parent metal.

It should be emphasized that experimentally obtained results imply the necessity for modification of procedures and recommendations, together with regulatory norms and standards that regulate design of the important welded constructions. The contemporary methods of welded construction calculation and design are based on relations for estimation of stresses. In that way, the estimated stresses remain within the critical limits during the project development, below the permissible stresses that are guaranteeing the integrity and functioning of the welded constructions. Estimated stresses in welded constructions demonstrate a high level of accordance with the real stresses obtained experimentally, due to the increasingly complex mathematical apparatus and application of the modern computer techniques. However, the modern materials demonstrate mechanical characteristics that substantially differ from material mechanical characteristics that were a basis for developing the mathematical models and algorithms for stress estimation in welded constructions. The full advantage of application of modern materials, such as the HSLA steels, can be achieved only if a specific nature of those materials is taken into account during the welded constructions design.

Experimentally obtained results correspond to the relevant literature sources, such as Pamnani et al. [28], Nathan et al. [29], Haribalaji, Boopathi and Balamurugan [30], Oyyuaravelu [31], and Çağırıcı et al. [32]. However, research presented in this paper is a deeper qualitative analysis of the welding procedure’s influence on fracture toughness of the HSLA steels. Sensitivity of those steels to the heat input additionally complicates this analysis and causes that only experimentally obtained results can be taken as fully relevant. The impact toughness of welded joints of the HSLA steels, which was the focus of research presented in this paper, is significantly less discussed in literature. On the other hand, level of application of this steel grade shows continual increase. Modern materials, used for the welded constructions, are characterized by difficult, even conditional weldability, especially from the aspect of obtaining favorable microstructure in the specific zones of the welded joints. The contemporary referent literature sources discuss the impact only of certain parameters on microstructure and only in certain zones of the weld. From practical aspects of application in the welded constructions’ design, it is more convenient to establish the correlation between the microstructure parameters and mechanical characteristics of the welded joints.

## Figures and Tables

**Table 1:** Chemical composition of S690 steel

Content of Chemical Elements, %													
C	Mn	Si	P	S	Cr	Mo	Ni	V	B	Cu	Ti	N	Nb
0.2	1.5	0.6	0.02	0.01	0.7	0.7	2.0	0.09	0.005	0.3	0.04	0.01	0.04

**Table 2:** Welding parameters and related consumables

Parameter	MMA	MAG
Consumables	INOX B 18/8/6; Shielded electrode diameter Ø 3.25 mm	MIG 75; Electrode wire diameter Ø 1.2 mm
Welding current, I_z_	I_z1_ ≈ 20 A	I_z1_, I_z2_, I_z3_ ≈ 250 A
Work pressure, U_z_	U_z1_ ≈ 24 V	U_z1_, U_z2_, U_z3_ ≈ 25V
Welding speed, v_z_	v_z1_ ≈ 0.2 cm/s	v_z1_, v_z2_, v_z3_ ≈ 0.35 cm/s
Fatigue energy, q_l_	q_l1_ ≈ 12 kJ/cm	q_l1_, q_l2_, q_l3_ ≈ 15 kJ/cm
Welding depth,	_1_ ≈ 1.8 mm	_1_, _2_,_3_ ≈ 1.9 mm
Wire handling speed	−	8 m/min
Protective gas	−	Gas compound M21-82% Ar + 18% CO_2_
Protective gas flow	−	14 l/min

**Table 3:** Chemical composition and mechanical characteristics of used filler materials

Type	Chemical Composition, %						Mechanical Characteristics			
	C	Si	Mn	Cr	Ni	Mo	R_m_MPa	R_p0,2_MPa	A_5_%	KVJ
INOX B18/8/6	0.12	0.8	7	19	9	−	590 to 690	> 350	> 40	> 60 (+ 20 °C)
MIG 75	0.08	0.6	1.7	0.25	1.5	0.5	770 to 940	> 690	> 17	> 47 (– 40 °C)
